# Incidence and Risk Factors for Surgical Site Infection in Ankle Fractures: An Observational Study of 480 Patients in Sweden

**DOI:** 10.3390/jcm12206464

**Published:** 2023-10-11

**Authors:** Johanna Bergström, Emilia Möller Rydberg, David Wennergren, Karin Svensson Malchau

**Affiliations:** 1 Institute of Clinical Sciences, Sahlgrenska Academy, University of Gothenburg, 405 30 Gothenburg, Sweden; emilia.rydberg@vgregion.se (E.M.R.); david.wennergren@vgregion.se (D.W.); karin.am.svensson@vgregion.se (K.S.M.); 2 Department of Orthopaedics, Sahlgrenska University Hospital, 413 80 Gothenburg, Sweden

**Keywords:** surgical site infection, fracture related infection, ankle fracture

## Abstract

Introduction: Surgical site infection (SSI) is a frequently reported complication after ankle fracture surgery. To our knowledge, no study has been conducted on its incidence in Sweden. The present study aimed to determine the incidence of, risk factors for, and most common causative pathogen of SSI. Methods: Patients who underwent primary surgery for an ankle fracture between 1 September 2017 and 31 August 2019 at the Sahlgrenska University Hospital were identified. Data on potential SSI risk factors and clinical outcome (infected/non-infected) were retrieved from medical records. Cox regression analysis and descriptive statistics were used. Results: Of the 480 reviewed patients, 49 developed SSI (10.2%), of which 35 (7.3%) were superficial and 14 (2.9%) were deep. Open fractures (*p* < 0.001) and age (*p* = 0.016) were statistically significant risk factors for SSI in the univariate analysis. In the multivariable analysis, only open fracture was statistically significant (HR = 3.0; 95% C.I. = 1.3–6.9, *p* = 0.013). Cases of *Staphylococcus aureus* (*S. aureus*) were most common (*n* = 12, 24.5%). Methicillin resistance was uncommon (*n* = 2, 4.1%). Conclusions: An incidence of 10.2% was established, which is comparable to international findings. Infection monitoring is an important part of tackling the global challenge of antibiotic resistance. Future prospective studies to further establish risk factors are warranted to decrease the incidence of SSI.

## 1. Introduction

Ankle fractures are the third most common type of fracture in Sweden, with an incidence of 127 per 100,000 person-years [1]. The Sahlgrenska University Hospital (SU) alone treats approximately 700 ankle fractures annually [2]. One of the most common complications after ankle fracture surgery is surgical site infection (SSI) [3], with an incidence of between 1 and 28% [3,4,5,6,7,8,9,10,11,12,13,14,15,16,17,18]. The increase of antibiotic resistance and its recognition as one of the greatest threats to global health [19] validates the importance of SSI research. Within ankle fracture surgery alone, methicillin-resistant *Staphylococcus aureus* (MRSA) causes 5.8–44% of SSIs [8,14,15,16,18], warranting the need for both infection monitoring and new strategies for infection prevention.

SSI is associated with increased morbidity and mortality, and high healthcare-related costs. The costs for a patient with a SSI can be up to double that of a non-infected patient [20]. Furthermore, SSIs have negative psychological, social and economic effects on patients due to long-lasting pain, isolation and insecurity [21], leading to a significant reduction in their quality of life [22]. A cornerstone of infection prevention is patient selection, i.e., assessing the risk of whether a patient may develop an infection. However, depending on the severity of the fracture, conservative treatment is sometimes not an option, or not sufficient. There are a number of suggested risk factors for SSI to be considered. For ankle fracture surgery, advanced age [4,5,9,16,23]; diabetes mellitus [4,6,11,12,23]; smoking tobacco [9,10,12,14,17]; ASA (American Society of Anaesthesiologists Physical Status) class 3–4 [4,11,23]; the presence of an open fracture [9,11,16]; and obesity [4,12,15,16] have been highlighted as patient-related risk factors. Moreover, as SSIs are multifactorial, other non-patient-specific factors need consideration, such as surgical details in the form of prolonged duration of surgery [9,10,15,24]; delay of surgery [13,25,26]; and the surgeons’ level of experience [15]. Patient selection and optimisation of surgery can be difficult, yet it is of great importance to increase the knowledge of which factors should be considered to improve infection prevention.

To the authors’ knowledge, no study on the incidence and risk factors for SSI after ankle fracture surgery has been conducted in Sweden. The aim of this study was to investigate the incidence of SSI after ankle fracture surgery. A secondary aim was to identify risk factors for SSI following ankle fracture surgery and to determine the most common pathogen causing these infections. The hypothesis was that the incidence of SSI after ankle facture surgery in Sweden is 1–28%.

## 2. Materials and Methods

This is a retrospective observational study conducted at SU in Gothenburg, Sweden. All patients ≥18 years of age who were diagnosed with an ankle fracture between 1 September 2017 and 31 August 2019 and underwent primary surgery were identified using the Swedish Fracture Register (SFR). Only patients with a Swedish personal identity number who experienced a fracture in Sweden are registered in the SFR.

The SFR is a Swedish national quality register started in 2011 [27]. Data, such as fracture classification, injury date and treatment details, are manually registered in SFR by treating physicians. When controlled against the National Patient Register in Sweden, the SFR has a completeness of approximately 70–90% for most of the orthopaedic departments [28].

Data on patient-, injury- and surgery-related variables were collected by reviewing medical records and the local surgery database at the hospital. Data on microbiology were retrieved from microbiological records. Exclusion criteria were <18 years of age, bilateral ankle fractures and residence in a city other than Gothenburg, Sweden. Records were reviewed on 9 September 2021.

All patients were given prophylactic intravenous antibiotics—Cloxacillin 2 g or, if allergic, Clindamycin 600 mg—30 min before surgery according to hospital routine. If an open fracture was present, antibiotic treatment was given at the emergency department. Patients were followed up three weeks post operation for wound status and suture removal, and additionally at six weeks post operation according to local routine. Data on signs of infection, positive microbiological cultures, use of antibiotics and re-operations were retrieved from medical records.

### 2.1. Definitions

The definition of SSI was assessed though a modified version of Metsemakers et al.’s definition of suggestive criteria for fracture-related infection (FRI) [29]. Infection status (infected/non-infected) was recorded within one year of the index surgery. SSI was defined based on wound status, antibiotic use, microbiological findings and need for reoperation, and further classified as superficial or deep (Table 1). Wound status at follow-up was classified according to the physician’s assessment: normal healing, delayed healing without suspicion of SSI, and SSI. Delayed healing was defined as a wound not healed at the follow-up six weeks post operation. Patients were excluded from the SSI group if the suspicion of infection was discarded at a later follow-up and treatment with antibiotics was ceased. For reoperations, SSI was ruled out if no intraoperative signs of infection were present and antibiotics were discontinued postoperatively.

Microbiological culture samples were defined as negative if no bacterial growth was present. Positive samples were categorised as monomicrobial, or polymicrobial if more than one species or strain was found. Cultures showing mixed Gram-positive flora were considered as a separate group due to their low clinical significance.

Surgeon experience was defined as resident, specialist, or consultant level. For analysis, only the main surgeon’s level of experience was included in cases where there was more than one surgeon present during the operation.

### 2.2. Statistical Analysis

Means with standard deviation (SD) and medians with interquartile range (IQR) were used for descriptive statistics. To compare the non-infected and infected groups, the chi-square or Fisher’s exact test were used for statistical evaluation of categorical variables, and the Mann–Whitney U test was used for continuous variables. A multivariable logistic regression analysis (Cox regression) was performed to further analyse risk factors for SSI. The significance level was set at *p*-value < 0.05. Statistical analysis was conducted in IBM SPSS version 26.

## 3. Results

### 3.1. Study Cohort

**Figure 1:**
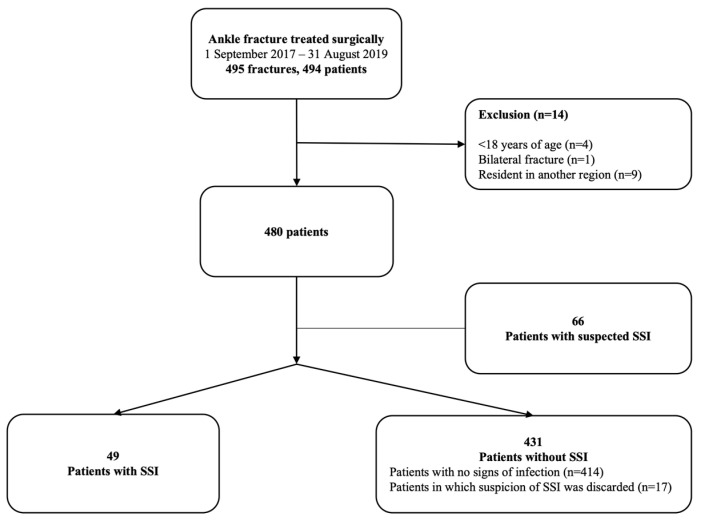
Flow chart of patient inclusion. Abbreviation: SSI, surgical site infection.

During the study period, 494 patients underwent primary surgery for an ankle fracture; of these, 14 patients were excluded from the study (9 patients resided in another city and were lost to follow-up, 4 patients were <18 years old and 1 patient had bilateral ankle fractures). In total, 480 patients were included (Figure 1).

Of the 480 included patients, the median age was 55 years (IQR: 29), BMI was 27.0 (IQR: 6.5) and the majority of patients were female (59.6%). Further characteristics of the study population are presented in Table 2. Within one year of surgery, seven patients died, whereof three experienced a SSI. However, no death was caused by a SSI.

### 3.2. Patients with a SSI

Of the 480 patients, 49 (10.2%) developed a SSI. The rate of superficial SSI was 7.3%, and for deep SSI, 2.9%. Of the 449 patients who had closed fractures, 39 (8.7%) developed a SSI; and of the 31 patients with an open fracture, 10 (32.3%) developed a SSI (Table 3).

Infected patients were older than non-infected patients (*p* = 0.016) and had a higher rate of open fractures (*p* < 0.001) (Table 4). Surgical characteristics did not statistically significantly differ between the two groups, although the infected group had a higher proportion of patients who waited more than 72 h for surgery and the non-infected group were, to a greater extent, operated on in ORs with laminar airflow (LAF).

The infected group had a longer hospital stay at the initial admission (*p* = 0.037) and a greater number of follow-ups (*p* < 0.001) compared to the non-infected group. The median length of antibiotic treatment of the infected patients was 10 days (IQR; 20, range 7–164). Patients who underwent reoperation due to infection had one to four reoperations before infection resolution.

### 3.3. Microbiological Findings in the Infected Patients

Of the 49 infected cases, 33 (67.3%) were sampled with microbiological counts and 16 (32.7%) had no samples available. Of the 33 sampled cases, 26 (78.8%) were positive for bacterial growth, making the incidence of SSI verified with cultures 5.4%. A total of 23 patients with SSI had either growth of mixed Gram-negative bacteria, no growth, or no samples available. There were 18 monomicrobial infections and 8 polymicrobial infections. *S. aureus* was the most common monomicrobial pathogen and was identified in 12 patients (24.5%). Two infections (4.1%) were caused by MRSA, whereof one was monomicrobial and one was polymicrobial. Further microbiological characteristics of the infected patients are presented in Table 5.

### 3.4. Risk factors for SSI

In the univariate analysis (Table 4), infected patients were older than non-infected patients (*p* = 0.016) and had a higher rate of open fractures (*p* = <0.001). However, in the multivariable analysis, only the presence of an open fracture was a statistically significant risk factor, with a threefold greater risk of SSI compared to patients with a closed fracture (HR = 3.0, 95% C.I. = 1.3–6.9, *p* = 0.013) (Table 6).

## 4. Discussion

This retrospective observational study of 480 patients with a surgically treated ankle fracture reveals a 10.2% incidence of SSI. Presence of an open fracture implied a threefold greater risk of SSI. The most common causative pathogen was *S. aureus*, and there was a low incidence of MRSA (4.1% of the infected patients).

The overall rate of infection (10.2%), with rates of superficial SSI at 7.3% and deep SSI at 2.9%, was comparable with previous findings of overall rates between 1 and 28% [3,4,5,6,7,8,9,10,11,12,13,14,15,16,17,18], rates of superficial SSI at 2.1–14.0% [8,17] and rates of deep SSI at 0.8–6.8% [8,10]. Differences in incidence rate may depend on the definition of SSI used, study design and inclusion criteria, as well as the country and level of hospital. The infection rate presented in the current study could be an underestimate, as some patients may seek care in the primary health care sector for wound problems that hence remain unknown to us. However, an overestimation may be more likely, due to the broad inclusion criteria of superficial infection, where some of the included cases may be a result of antibiotic overuse. Nonetheless, the incidence of SSI in the current study mirrors the clinical reality of patients treated for suspected SSI at our department.

*S. aureus* is a common pathogen in orthopaedic infections, which is confirmed in the current study with a rate of 24.5%. Similar incidence rates have been found in studies from both Finland (32.8%) [10] and China (17.8%) [8]. In the current study, the rate of MRSA (*n* = 2, 4.1%) is low in comparison to existing literature where rates of 5.8–44% have been reported [8,14,15,16,18]. This finding was not surprising though as Sweden, in general, has a low rate of MRSA compared to other countries [30]. Similar studies should be conducted in the future to continue the surveillance of MRSA and other drug-resistant bacteria.

In the current study, open fractures implied a threefold increased risk for SSI. The presence of an open fracture has previously been established as an independent risk factor for SSI [9,11,16,31]. The most obvious explanation may be that the fracture punctures the skin, at which point bacteria can contaminate the tissues. Several other patient-related risk factors have been reported to contribute to SSI risk, such as advanced age; smoking tobacco; diabetes mellitus; male sex; high ASA class (3–4); and obesity [4,5,6,9,10,11,12,14,15,16,17,23]. However, the current study could not confirm this, which may be due to the size of the cohort, but also due to selection bias. Patients with well-known risk factors, such as advanced age, diabetes or smoking, may receive non-surgical treatment instead of surgery to a greater extent, due to a higher risk of infection. This selection might contribute to the idea that that these well-known risk factors do not stand out as risk factors in the analysis of this retrospective study. Interestingly, Schade et al. reported that only insulin-dependent diabetes was a risk factor for SSI, as opposed to non-insulin-dependent diabetes [23]. Similarly, Neuman et al. stated that there was an increased risk of complication postoperatively in posterior malleolus fractures when insulin-dependent diabetes mellitus was present ref. [32]. The current data was not controlled for this possible confounding. No association was found between SSI and surgical characteristics, such as delay of surgery from admission, use of temporary external fixation, duration of surgery, or use of LAF in the OR. Again, this might be due to the size of our study population, as both delay of surgery and duration of surgery have previously been reported as risk factors for SSI [9,10,13,15,24,25,26].

This study has limitations. Firstly, the retrospective design and the reliance on data assessed through medical records is a limitation. Medical records as a data source can compromise data accuracy, due to the lack of a standardised way to document signs of infection. Details of the extent of soft tissue damage and the classifications of open fractures were not stated in the medical records; therefore, we were not able to obtain this type of information. The retrospective design did not allow us to classify the surgical wound according to current definitions of SSI, such as the CDC [33] or FRI classifications [29]. Moreover, it is of great importance to use the available diagnostic criteria of SSI when possible, to reduce the risk of overuse of antibiotics. In 23 cases, the diagnosis of SSI can be questioned as the infection was not verified with cultures, making the incidence of SSI verified with cultures 5.4% (26/480). Notably, in three cases, antibiotics had been started prior to sampling which could have led to false negative results. However, in all included SSI cases, a clinical suspicion of infection was confirmed by a physician in an orthopaedic department, and despite the difficulties of SSI diagnostics, the staff are experienced in this field. The incidence found in the present study reflects the clinical reality of treated SSI. Current SSI definitions, such as the CDC’s [33], have their limitations as the depth of the SSI needs to be evaluated, which requires a reopening of the incision. In fracture surgery, this exposes the fracture and hardware, which risks bacterial contamination. However, to optimise the possibility of SSI classification, a prospective study is warranted. Further, all cases defined as SSI resulted in the use of antibiotics and additional follow-ups, which increase costs, making the chosen definition of SSI clinically meaningful. Patients treated with prolonged antibiotics, who need multiple hospital visits and experience local symptoms, can be assumed to suffer to some extent from this [21], making these cases important to acknowledge.

Compared to larger studies on more than a thousand patients [8,10], the cohort of the current study is rather small; this might be the reason why this study did not reach statistical significance for the tested variables. A rule of thumb for Cox regression is the “one in ten rule” which implies that there is a need for a minimum of ten outcomes per variable [34]. According to this rule, there is a risk of overfit in the present study. However, the “one in ten rule” has been questioned and may be too conservative; instead, five to nine outcomes per variable has been suggested as a valid option [35].

Despite its limitations, the current study was able to gather data from one of Scandinavia’s largest orthopaedic departments. The standardised times for follow-up at the hospital allowed for consistency for all patients regarding wound examination. We also had access to several variables which, combined, could be used to determine the presence of SSI. Furthermore, to the authors’ knowledge, the current study was the first in Sweden to assess the incidence of SSI after surgically treated ankle fractures. Additionally, the causative pathogens and the rate of MRSA, as well as risk factors of SSI, were presented. This knowledge is important for further development of safer surgery and infection prevention.

## 5. Conclusions

In the present study, surgically treated ankle fractures had a 10.2% risk of SSI. *S. aureus* was the most common pathogen and the rate of MRSA was, as expected, low compared to international results. Patients with an open fracture had a three times higher risk of SSI, which should be considered when managing these injuries. The current study contributes to infection monitoring, which is a crucial part of the global fight against antibiotic resistance. However, there is still a need for large, prospective multicentre studies to further establish risk factors and thereby identify possible infection preventative measures.

## Figures and Tables

**Table 1:** The definition of surgical site infection used in the current study.

Surgical Site Infection:	
Superficial	If ≥1 of the following was present within a year of index surgery: Clinical suspicion of infection Postoperative antibiotics due to signs of infection (swelling, redness, warmth or pain) Wound swab with a positive microbiological count
Deep	If a reoperation, due to clinical signs of infection, occurred within a year of index surgery and ≥1 of the following was present: A positive microbiological count The continued use of antibiotics postoperatively

**Table 2:** Characteristics of the study population (*n* = 480).

Characteristics	Median (IQR, Range)
Age	55 (29, 18–93)
Female sex, *n* (%)	286 (59.6)
BMI	27.0 (6.5, 17.7–63.2)
Time until surgery (h)	72.5 (131, 3–1085)
Duration of surgery (min)	88 (48, 9–300)
Length of stay (days)	3 (5, 0–33)

Abbreviations: BMI, body mass index; h, hours; IQR, interquartile range; min, minutes; *n*, number.

**Table 3:** Infection incidence according to fracture type.

Type of Fracture	Infection Incidence—*n* (%)
All	49 (10.2)
Superficial	35 (7.3)
Deep	14 (2.9)
Closed	39 (8.7)
Superficial	28 (6.2)
Deep	11 (2.4)
Open	10 (32.3)
Superficial	7 (22.6)
Deep	3 (9.7)

**Table 4:** Characteristics of the infected and non-infected patients with univariate analyses.

Characteristics	Infected—*n* (%) 49 (100)	Non-Infected—*n* (%) 431 (100)	*p*-Value
Age ^+^, median (IQR, range)	61 (25, 21–93)	55 (29, 18–91)	0.016
Female sex	30 (61)	256 (59)	0.805
Diabetes *	6 (12)	36 (8)	0.419
Smoking	12 (24)	68 (16)	0.154
ASA 1–2	41 (84)	386 (90)	0.213
ASA 3–4	8 (16)	45 (10)	
BMI *:			0.374
<18.5	1 (2)	1 (0)	
18.5–24.9	15 (31)	127 (29)	
25–30	18 (38)	162 (38)	
>30	14 (29)	123 (29)	
Open fracture *	10 (20)	21 (5)	<0.001
Time until surgery <24 h	8 (16)	79 (18)	0.632
24-72 h	12 (24)	131 (30)	
>72 h	27 (55)	212 (49)	
Duration of surgery >90 min	25 (51)	193 (45)	0.601
LAF	24 (49)	247 (57)	0.228
Temporary external fixation *	7 (14)	35 (8)	0.176
Head surgeon:			0.249
Resident	15 (31)	165 (38)	
Specialist	12 (25)	124 (29)	
Consultant	22 (45)	141 (33)	
Length of stay (days) ^+^, median (IQR, range)	4 (8, 0–20)	3 (5, 0–33)	0.037
No. of follow-ups ^+^, median (IQR, range)	5 (7, 1–26)	3 (2, 0–24)	<0.001

No. of follow-ups: number of outpatient visits to a doctor after index surgery, either in the emergency room or orthopaedic department. Statistical analysis: Pearson’s chi-square, Fisher’s exact test *, Mann–Whitney U^+^. Abbreviations: ASA, American Society of Anaesthesiologists; BMI, body mass index; h, hours; LAF, laminar air flow; LOS, length of stay; min, minutes; n, number; No, number.

**Table 5:** Microbiological characteristics of infected patients.

	Frequency—*n* (%) 49 (100)
Positive microbiological count	26 (53)
Monomicrobial infections	18 (37)
*S. aureus*	12 (24)
MRSA	1 (2)
CoNS	1 (2)
*Corynebacterium striatum*	1 (2)
Acinetobacter	1 (2)
Group G β-hemolytic streptococci	1 (2)
Escherichia coli	1 (2)
Polymicrobial infections	8 (16)
*S. aureus* + *Enterobacter cloacae*	1 (2)
CoNS + MRSA	1 (2)
CoNS + Clostridium	1 (2)
CoNS + Pseudomonas	1 (2)
CoNS + Enterobacter cloacae	2 (4)
*Corynebacterium striatum* + *Proteus vulgaris*/penneri	1 (2)
*Corynebacterium striatum* + *Peptoniphilus*	1 (2)
Mixed Gram-positive bacteria	4 (8)
Negative count	3 (6)
No samples available	16 (33)

Abbreviations: CoNS, Coagulase-negative staphylococci; MRSA, Methicillin-resistant *Staphylococcus aureus*; *n*, number; *S. aureus*, *Staphylococcus aureus*.

**Table 6:** Cox regression analysis of risk factors for SSI.

Variables	Hazard Ratio	95% C.I.	*p*-Value
Age *	1.016	1.00–1.36	0.122
Male sex	1.072	0.56–2.05	0.834
Diabetes	1.407	0.53–3.71	0.490
Smoking	1.232	0.58–2.64	0.591
ASA 3–4	0.866	0.33–2.56	0.768
BMI *	0.991	0.93–1.06	0.789
Open	2.960	1.26–6.96	0.013
Time from ER to surgery *	1.001	1.00–1.00	0.227
Duration of surgery *	1.000	1.00–1.00	0.990
OR without LAF	1.221	0.65–2.28	0.532
External fixation	1.045	0.39–2.80	0.930
Head surgeon: Resident	0.993	0.45–2.18	0.986
Specialist	Ref.		0.797
Consultant	1.245	0.58–2.69	0.576

String variables *. Abbreviations: ER, emergency room; ASA, American Society of Anaesthesiologists; BMI, body mass index; LAF, laminar air flow; OR, operating room; Ref., reference.

## Data Availability

The datasets generated and analysed during the current study are available from the corresponding author on reasonable request.
